# Salivary Markers Responses in the Post-Exercise and Recovery Period: A Systematic Review

**DOI:** 10.3390/sports11070137

**Published:** 2023-07-18

**Authors:** Rafael Santos Neves, Marco Antônio Rabelo da Silva, Mônica A. C. de Rezende, Adriana Caldo-Silva, João Pinheiro, Amândio M. C. Santos

**Affiliations:** 1 Faculty of Sports Science and Physical Education, University of Coimbra, 3040-248 Coimbra, Portugalamandiosantos@fcdef.uc.pt (A.M.C.S.); 2 Faculty of Physical Education, University of Amazônia, Santarém 68040-255, Brazil; 3 Faculty of Physical Education, Federal University of Pará, Belém 66075-110, Brazil; 4 Research Centre for Sport and Physical Activity, University of Coimbra, 3040-248 Coimbra, Portugal; 5 Faculty of Medicine, University of Coimbra, 3000-548 Coimbra, Portugal

**Keywords:** biomarkers, sports performance, physical activity, hormonal responses

## Abstract

The use of saliva to monitor immune and hormonal responses in training, competitions, and during recovery is an easy and non-invasive alternative means of collecting samples compared to serum collection. Saliva can provide insight into a number of interesting biomarkers such as cortisol, testosterone, immunoglobulins, alpha-amylase, and melatonin, among others. High-intensity and exhaustive exercises, such as training or competition, provide variations in immune, protein and hormonal markers. An adequate recovery period, calming down, and recovery methods can contribute to a fast normalization of these markers, decreasing illness, as well as the likelihood of overtraining and injuries, but their effectiveness is still inconclusive. The aim of this review was to investigate the evidence of salivary markers in post-exhaustive exercise during the recovery period. This study is a systematic review from three electronic databases with studies from 2011 to 2021 within healthy humans. The search found 213 studies, and after applying the inclusion and exclusion criteria, while excluding duplicated studies, 14 studies were included in this review. The most cited salivary markers were cortisol and testosterone, as well as their ratio, alpha-amylase and IgA. Half of the studies applied a variety of recovery methods that showed controversial results over salivary markers’ impact. However, they showed an impact on the markers from the exercise, which was still dependent on exercise intensity, methodology, and duration.

## 1. Introduction

High-intensity exercises during training sessions and competitions lead to muscle fatigue, limit performance, and provide increased risks of overtraining, injuries, and immunological, inflammatory, and hormonal changes [1,2,3]. Studies have shown increases in illness with an imbalance in training and recovery, but these findings are uncertain because there is no agreement on the immunodepression mechanism in the response to recovery [4,5].

In recent years, saliva has been used as an alternative to serum for the analysis of immune markers, hormones, steroids, non-steroids and protein compounds, being a non-invasive and stress-free choice, despite the concentrations in saliva differing from those in serum across some markers. Saliva samples have shown good correlation to analyses and can be collected in the laboratory as well as in the field frequently, rapidly and without medical training [6,7,8,9]. The immunologic system’s first line of defense, the salivary mucosa, was one of the first areas studied due to the suppression of the immune system in response to exercise and related diseases, although there is still no conclusive association [10,11]. 

The search for better performance and the prevention of diseases and overtraining, as well as the separation of immune and hormonal responses, so that there is no change in performance between training and competitions, have led to the use of physical therapies and nutritional supplementation as recovery methods, both in acute and chronic cases.

This systematic review aimed to investigate the evidence available in the literature around salivary markers that have been used and their impacts on post-exhaustive exercise in the acute recovery period, and the variation that some recovery methods can apply to these salivary markers. Considering the costs associated with the analysis of salivary markers, this study may prove relevant in order to not repeat certain unnecessary analyses in which there is already evidence confirming the responses of these salivary markers in fatiguing exercises and the recovery period, as well as evidence showing those markers that are more important regarding the relevant issues.

## 2. Materials and Methods

### 2.1. Data Sources

This systematic review was completed in accordance with the recommendations of the *Cochrane Handbook of Systematic Review*, and has already been registered on PROSPERO (CRD42021240469). A computerized literature search was performed using three online databases: PubMed (Medline), EBSCO (SportDiscus) and SCIELO. The following keywords and queries were used to find relevant papers: “saliva markers OR salivary markers OR saliva biomarkers OR salivary biomarkers AND exercise recovery”.

### 2.2. Inclusion and Exclusion Criteria

Studies that met eligibility criteria based on PICOS (population, intervention, control, outcomes, and study design) were included if they used saliva, or salivary markers, and whether these markers were also measured in acute moments of recovery after exercise, even without assigning a recovery method, in which case passive recovery was assumed. Measurements in acute recovery with at least one measurement between 30 min and 48 h were accepted. Necessarily, there must have been an application of physical exercise, whether in a controlled laboratory environment, physical training, or competition, even if simulated. Studies were excluded when performed with animal samples or with humans with diseases, or if there was no post-recovery measurement of at least 30 min. Studies with only pre- and immediately post-recovery measurements were excluded.

### 2.3. Study Selection

**Figure 1:**
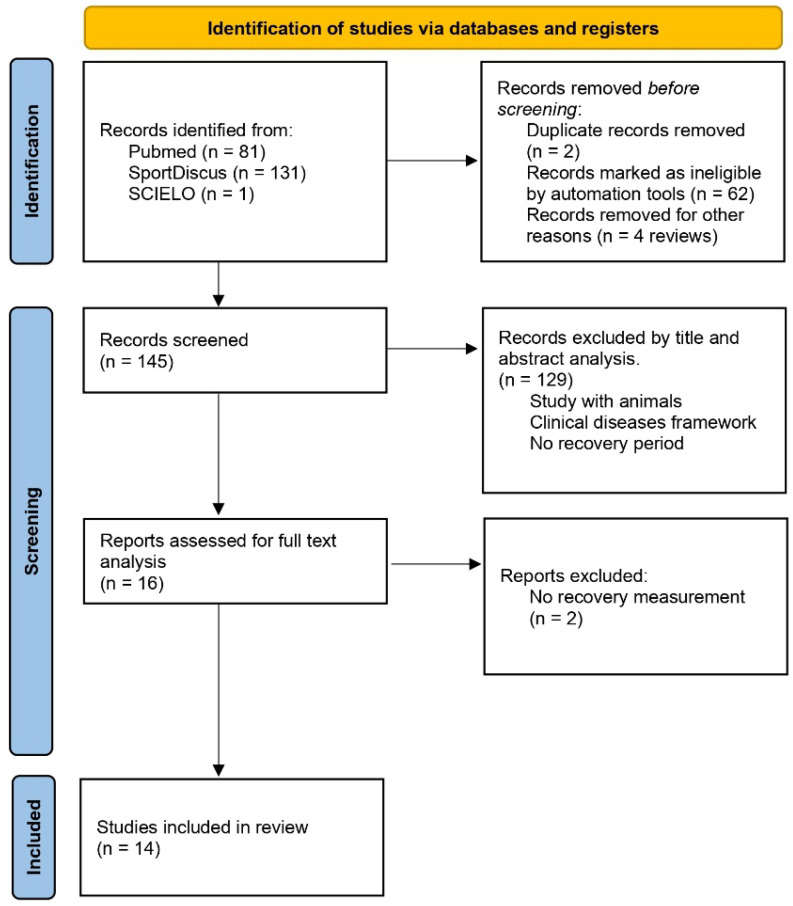
Summary of search strategy and selection process.

The search was conducted across many types of studies, including clinical trials, controlled clinical trials and randomized controlled trials, and 81 studies were found in the PubMed database, 131 studies in SportDiscus and just one in SCIELO (Figure 1). The study found in the SCIELO database was excluded because it was related to dental and oral hygiene, as well as being a review study. The search criteria of studies in English, concerning humans only, and having been conducted in the past 10 years (1 January 2011 to 10 December 2021) were applied. Duplicate studies, found in more than one database, were considered as a single study (duplicate deletion), and evaluation through study titles and abstracts was conducted to exclude those with samples not consistent with the research; in the end, a total of 16 studies were selected.

After reading and analyzing the full text, one study was excluded because there was no recovery moment, only pre- and post-measurement, as well as another study that followed up on the responses to training for 12 weeks. After reading the full texts, 14 studies were included in this systematic review. The PEDro quality scale was used to perform an internal validity analysis of each study.

## 3. Results

Of the 213 studies found in the three databases, and after applying search criteria and careful analysis of titles, abstracts, and full texts, 14 studies were selected for this systematic review. Table 1 presents the PEDro scale analysis [12]. The eligibility criteria (non-internal validity PEDro item) were presented in 86% (*n* = 12) of studies, and statistics information to analysis and interpretation with group comparisons were present in 72% (*n* = 10) and 100%, respectively, showing points and variability measures. For the internal validity items, just two studies used random and/or concealed allocation to groups. Baseline data were presented in 93% (*n* = 13), and all studies (100%) showed adequacy of follow-up. In 78% (*n* = 11) of studies subjects received an intention to treat analysis, and a comparison between groups, exercises methodologies, or effect time was present in 71% (*n* = 10). No one study used a blinded methodology, even for volunteers, therapists, trainers, or evaluators.

### 3.1. Samples and Ages

The characteristics of the volunteers, previous physical stress activity and measured variables are described in Table 2 (analysis of included articles). In a total of 226 volunteers, just thirteen were women. However, gender was not observed in one study [13]. An apparent uniformity was found in relation to the volunteers age range, ranging from 18 to 30 years old, with exceptions in four studies that differ in ages: one with a sample age around 14 years old [14], two other studies [13,15] with a sample age over 30 years and a fourth study [16] that did not mention sample age. The volunteers from eleven studies were well trained athletes from 10 different sports; rugby was the most common and included in five studies [16,17,18,19,20]. One study [21] just cited that the volunteers were trained, without a sport mentioned, and two other studies with healthy and active volunteers [6,22].

### 3.2. Exercises Interventions

The exercise procedures used to generate physiological stressors (fatigue, muscle damage or exhaustion) varied and included tests in the laboratory or field, official competitions or simulations, and physical training. Five studies were found with laboratory tests using isokinetic [23] and cycle ergometers [21,22,24] and field tests with sprints [20,25]. Three studies used training sessions as exercise procedures where one [10] of them analyzed the influence of the prescription training order, and another one [14] a high-intensity exercise protocol, with cycles composed of a countermovement jump (CMJ) followed by a go-and-back 20 m sprint and another CMJ, as well a simulated competition with an MMA contest-preparation training session [13]. Official competitions were used as physiological stressors like rugby matches [16,18,19], a Taekwondo competition [14] and the 2008 London Marathon [15].

### 3.3. Saliva Collection Methods

Saliva can be collected, without medical training, non-invasive and non-stress, by two methods: whole saliva by passive droll, and using a cotton swabs, that promote minimal risk of gingival bleeding [4], as described by three studies in this review [14,15,24], or using a sterilized plastic tube [10,13,16,18,19,21,22,23,25]. Major studies described the unstimulated passive droll [6,13,14,16,22,24,25,26,27,28,29], but the time used to collected the saliva were not congruent. Two studies used a sample collection time of one minute [13,16], four used two minutes [14,20,22,25], one study used five minutes [30] and four did not describe how long the collection took [10,17,18,19], and three studies did not use time for collection but the volunteers needed to droll at least 1 mL [6,31] or 0.5 mL [21].

### 3.4. Variables

A saliva sample has a large variety of possibilities for variable analysis [1,9]. Cortisol, considered the main hormone for catabolic processes, is secreted as a consequence of intense and stressful exercises, and psychological stress, which with insufficient recovery, could cause immunosuppression [4,32,33]. A significant correlation between serum and saliva with cortisol has been reported [9,18], and could be a reason that 13 of 14 studies [10,13,14,15,16,17,18,19,20,21,22,23,25] used this stress hormone. Cortisol increased significatively post-game (*p* = 0.002) compared to pre-game in adolescent rugby players and normalized 17 h after [16], as well as in young adult rugby players at 12 h post-game with a significant time effect (F = 4.9; *p* = 0.01) but continued to be significantly higher 36 and 60 h after compared with the baseline [19]. Also evaluating young adult rugby players [18], cortisol was measured extensively (24 h and 30 min pre- and 30 min, 24, 48, 72, 96 and 120 h post-exercise), showing significant increases 30 min (*p* < 0.001) and 24 h (*p* < 0.000) post-exercise and was significantly lower at 96 h (*p* = 0.042) compared with 24 h pre-exercise, and also showing the psychological impact of anxiety with concentrations being significantly higher 30 min to 24 h pre-exercise (*p* = 0.043). When they analyzed cortisol between moments and gender [14], the main effects were for gender (F = 8.74; *p* = 0.01) and sampling (F = 0.45; *p* < 0.0001) with lower concentrations from female athletes, despite the fact that in both genders, cortisol concentrations were significative higher after 30 min of recovery; however, after 90 min recovery concentrations were lower compared to pre-exercise. Also comparing moments [22] for intense exercise bouts on a cycle ergometer, cortisol increased significantly immediately after (*p* = 0.01) and returned to baseline concentrations after 30 min (*p* = 0.79) compared to pre-exercise. Comparing two different training prescription orders [34], significant time effects (F = 11.665; *p* = 0.000) but not interactions (F = 0.814; *p* = 0.494) were found, with similar results over time except for a significant difference, to both training orders, between 2 h post- and pre-exercise. However, when the responses were compared to different exercise intensities (40%, 60% and 80% of VO2max) [21], they only found significant differences with high intensity from pre- to post-exercise (105.3%; *p* = 0.005) and pre- to 30 min post-exercise (170.6%; *p* = 0.007), where a significant correlation between serum and salivary cortisol was found (r = 0.728; *p* = 0.001). When a recovery method was applied, cortisol did not change 2 h post-exercise using whole-body hyperthermia (WBH) but was lower in the control group with passive recovery [23]. Comparing external counterpulsation (ECP) with a control group (CG) using the same equipment without cuff inflation [17], a significant interaction was found (F = 4.07; *p* < 0.05), with higher concentrations in the CG at post- compared to pre-exercise and 24 h post (both *p* < 0.001) but in the ECP group, concentrations were only greater 24 h post (*p* < 0.0001) without a significant difference between pre- and immediately post-exercise. Evaluating performance athletes in the London marathon, where half of them used a cherry juice blend and half a placebo, no treatment or interaction effects were found; only a significant time effect was found, in both groups, for post-race compared to 24 h before the race (F = 26.291; *p* < 0.001) and this was restored after 24 h [15]. In MMA (Mixed Martial Arts) contest training [13], comparing cold water immersion (CWI) with passive recovery, significant increases were found pre- to immediately-post (*p* < 0.05) and 1 h post-training (*p* < 0.01), with elevated concentrations maintained until 2 h post (*p* = 0.06) and normalized after 24 h, and in the recovery methods comparison, they were similar in all moments except at 2 h post where passive recovery was significantly greater (*p* < 0.05). Still using cryotherapy, a whole-body cryotherapy was used for recovery but with no difference (F = 0.253; *p* = 0.859) compared to passive recovery, varying in time effect only (F = 13.998; *p* < 0.001) with significant differences at 2 h post (*p* = 0.003) and restored at 24 h [25]. Likewise, vascular occlusion did not differ from the control as a recovery method (*p* = 0.679), and sprints only decreased cortisol (F = 32.651; *p* < 0.001), which declined over time (F = 7.806; *p* < 0.001) and did not affect recovery (F = 0.640; *p* = 0.531) [20].

Testosterone variations have direct effects on body composition and skeletal muscle changes, improving muscle strength and protein anabolism and catabolism, among others [35]. The correlation between salivary and serum testosterone does not seem to differ in individuals at rest or after resistance exercise, although there are differences between gender and ages (pubertal development) [7]. In an analysis of the main effect of testosterone over time [19], significant differences were found (F = 3.34; *p* = 0.03), with an emphasis on the post-exercise comparisons of 12 (MD = 57.34; *p* < 0.05) and 36 h (MD = 41.31; *p* < 0.05) with the baseline, and normalization after 60 h (MD = 18.86). However, with intense exercise bouts on a cycle ergometer [22], a significant increase in testosterone (*p* = 0.001) was found immediately post-exercise, which remained elevated after 30 min (*p* < 0.001) compared to pre-exercise, with a significant time effect (F = 70.914; *p* < 0.001). When applied to two orders of training [34], a small-sided game (SSD) followed by resistance training (RES) and the other way around, both protocols had a significant time effect on testosterone (F = 5.471; *p* = 0.003), except at 2 h post-exercise for the protocol that started with SSD (*p* = 0.001), and there was a significant interaction between protocols (F = 5.196; *p* = 0.004), but with a greater elevation (*p* = 0.01; *d* = 0.73) post-exercise for the protocol that initiated with RES. In the three studies where a recovery method was applied, recoveries were found after external counterpulsation (ECP) [17], whole-body cryotherapy (WBC) [25] and thigh vascular occlusion (OCC) [20], when compared with a passive recovery or with a sham recovery. Significant effects for time were found in all studies (ECP: F = 15.02; *p* < 0.001/WBC: F = 6.275; *p* = 0.001/OCC: F = 20.127; *p* < 0.001), however when comparing recovery methods, ECP did not show differences (*p* > 0.05) between conditions, as well as OCC (*p* = 0.226), but WBC presented greater concentrations at 2 and 24 h post-exercise (*p* = 0.002).

The testosterone/cortisol concentration ratio (T:C) has been used as exercise stress index, an anabolic/catabolic indicator balance, where a decrease in T:C indicates an insufficient recovery after exercise, training, or competition, and may have negative influences on the repair of damaged skeletal muscle and lead to the development of overtraining syndrome [11,28,36]. The T:C was observed as a response to a comparison of two orders of training [10] without a recovery method, with a significant time effect (F = 15.333; *p* = 0.000) but no interaction (F = 0.877; *p* = 0.462) and with only a difference 2 h after exercise when the order that used the aerobic activity before resistance had a higher ratio (*p* = 0.001). When recovery methods were applied, ECP [17] shows a significant interaction (F = 4.54; *p* < 0.05) and higher results 24 h post, compared to immediately post-exercise for both groups (ECP: *p* < 0.05; *d* = 3.09/control group: *p* < 0.05; *d* = 0.31). However, they did not observe any interactions or significant differences between conditions and control groups, either by OCC (F = 0.299; *p* = 0.759; between recoveries: *p* = 0.421) [20] or by WBC [25], only with differences in time effect (OCC: F = 19.200; *p* < 0.001/WBC: F = 8.66; *p* = 0.001).

Alpha-amylase was analyzed in adolescents with no differences between gender but showed significant differences in the effect of time in relation to peak value at the end of the match and normalized after a recovery period of 30 min [14]. Likewise, with young men, a significant time effect was found when compared pre- and 24 h post-exercise to immediately post-exercise, but without differences when adjusted for secretion rate, however, significant differences were found in main effects for groups (control and external counter pulsation) in raw and secretion rates [17]. Produced as an innate defense support to IgA, alpha-amylase inhibits the growth of bacteria and attaches to mucosal surfaces, and with less attention, some studies have been shown increases in alpha-amylase concentrations [33].

Antibodies, also known as immunoglobulins, have the function of protecting the organism through a direct attack on the invading agent or by activating a complementary system so that it destroys the invader [37]. The IgA shows low concentration levels after exhaustive exercises or high intensity training sessions [37,38], but this is still dependent on sports modality, exercise intensity, duration, and practice continuity [30,39,40]. The results found in this review are not congruent. Of those who compared recovery methods, one study [15] did not find any time or interaction effect on IgA when compared to the placebo with cherry juice blend (*p* > 0.05), but found a time effect for both conditions (F = 7.560; *p* < 0.001), with a decrease post-exercise and normalization at 24 h post-exercise. In contrast, another study [17] found significance (F = 4.85; *p* < 0.05) to interactions effects and greater concentrations of IgA post-exercise compared to pre- and 24 h post-exercise (both *p* < 0.05; *d* = 1.26) using ECP as recovery, while the CG had no changes. Conversely, a study with senior rugby players [16] found no significant changes (409 ± 223 to 414 ± 255 µg.mL^−1^) between moments in IgA and IgA secretion rate. Immunoglobulin G (IgG), most abundant in blood but with lower quantities in saliva, was used in a comparison between a cherry juice blend and placebo as a recovery method for marathon runners [15], where no differences were found for time, treatment, or interaction effects. As the main antibody found in serum and tissues, with the exception of mucous membranes, IgG directly fights infectious agents [31].

In a single study [24], melatonin was used to analyze the effects of intense exercise, with and without cold water immersion (CWI) recovery, with no main effect for conditions (*p* = 0.96) or interaction of condition and time found (*p* = 0.84); however, melatonin increases in all conditions during the time after exercise with differences in the main effect (*p* < 0.001), even after 10 h of being awake. Sleep is important for recovery and skills acquisitions [26], and melatonin is a key hormone in the sleep-wake cycle which facilitates sleep onset, is barely visible during the day but elevated before and during sleep, is directly associated with human health and physical and cognitive performance. Melatonin production has been investigated for its potential role in increasing the frequency of training and competition [1,24,27,41].

## 4. Discussion

The selected studies mostly present a male sample, with only a total of 13 women. In addition to the low number of the female samples, it was not shown if there was control of the menstrual period. The study samples concentrated on healthy, trained volunteers and athletes, mostly young adults, with no analyzes on sedentary or older individuals, beyond 40 or 50 years, or the elderly.

Analyzes of biomarkers through saliva have been carried out due to a less invasive material collection profile, of lower cost and because there are already studies in which they have shown a good and significant correlation with the analyzes through peripheral blood collection, either with volunteers at rest or after resistance exercise [7,9,18].

There is an interesting variety of exercise interventions. Some with laboratory tests, where there is a greater control of intensity and movement, but sometimes without the reproducibility of sports mechanics [21,22,23,24]. Field tests, in turn, are closer to reality but make it difficult to control variables such as intensity [25,29], as well as the variations that may exist in training sessions [10,13,14] and competition simulations [15,16,18,19], even more so when observing the psychological responses of individual form. Intensity control is one of the factors that can directly influence the secretion levels of salivary markers, as well as the psychological impact during a competition, exacerbating changes in concentrations of these biomarkers.

Saliva collection has proven to be an effective, easy-to-perform, non-invasive and low-cost methodology, in addition to having a good correlation with blood collection. Most of the studies analyzed in this review used passive droll [10,13,16,18,19,21,22,23,25], where there may be a risk of gingival bleeding and contamination of the saliva sample [4], and only three studies used cotton swabs [14,15,24]. The difference in the preference for using a certain collection methodology may be due to the cost at which it is applied.

Saliva can provide a wide variety of biomarker analyses, but there seems to be a predilection for cortisol, as this was analyzed in 13 of the 14 studies. As the main growth hormone, it is described as highly susceptible to physical stress (exercise) and may be the cause of immunosuppression when its concentrations are not normalized [4,32,33]. Alterations in cortisol concentrations can be observed immediately after exercise, however there is a variation in the time taken for normalization, regardless of the application and type of recovery method, which can be influenced by the imposed exercise and its duration and intensity.

In testosterone, once again, there is a significant change after exercise and, as in cortisone, there is a variation in the time taken to regularize the baseline concentrations [35]. Only three studies applied recovery methods, comparing them to passive recovery, and their results showed little influence of recovery methods on testosterone concentrations. The variation, or divergence, of the results may be justified by the small sample used or by the diversity of sports practiced by the samples or the intensity of the exercise imposed [17,20,25].

Alpha-amylase has an increase in its concentrations when exposed to physical exercise. In adolescents and young adults, alpha-amylase levels seem to normalize in a short period of time, as well as showing a positive influence under the influence of DBS during the recovery period [10,17,20,25]. ECP also showed positive effects on IgA concentrations after 24 h since it also undergoes significant changes immediately after exercise [17]. However, such an impact was not observed in another recovery methodology with cherry juice blend ingestion [15].

The other markers (IgG and melatonin) did not show susceptibility to the recovery methods employed and only melatonin [24] was influenced by physical exercise. The small number of studies (1 each) and the low number of samples may not reflect the possible impacts that recovery methods have on biomarker concentrations. The intensity, even though it may seem strenuous exercises, may not have been enough for a significant increase in IgG secretion [15].

This study was limited to studies in the English language only, in three databases and three types of study (clinical trials, controlled clinical trials and randomized controlled trials) from the previous 10 years. However, advantages can be observed from the positive use of salivary markers, such as cortisol and testosterone, in their responses to exercise and recovery, demonstrating that they are applicable tools for evaluations in athletes and practitioners of physical exercise. A comparison between males and females was also not possible due to the small number of female volunteers evaluated in the studies compiled here.

## 5. Conclusions

It is correct to affirm that cortisol is the most used salivary biomarker during recovery periods. In the same way, this review observed a significant impact of exercise on the principal salivary biomarkers (cortisol and testosterone). However, the responses immediately after exercise for the other markers are still questionable since they were used in a small number of studies and with a restricted sample.

Approaching the question of the impact of recovery on the salivary biomarkers, there seems to be an acute impact to normalize their concentrations within a period of up to 24 h, but this remains inconclusive. The intensity and type of exercise could influence the responses. Another bias is the low number of studies and samples, and the impact of the use of a diversity of recovery methods on some biomarkers.

In short, salivary cortisol, testosterone, and alpha-amylase can be used to assess the impact of exercise, as well as responses to recovery methods, in addition to being easily accessible. But there are still doubts about the use of other salivary markers.

The diversity of recovery methods and methodologies, and the type, intensity, and duration of the physical stressor (exercise) probably influenced the salivary markers’ responses, as well as the psychological stress in competitions. That shows the necessity for a large study with more volunteers, different and cheap recovery methods, and a congruent methodology for reliability, which is similar to training sessions and competitions bouts that even physically active non-athletes can use day by day to improve their health and avoid the possibility of overtraining, adverse symptoms, or upper respiratory tract illnesses, and avoid prolonged absences from the practice of physical exercise.

## 6. Practical Applications

This study pointed out the main biomarkers used in assessing the impact of exercises and recoveries. The use of salivary markers leads to a lower cost for the studies as well as easier access because it is not invasive. However, it shows the need for a greater number of studies to analyze the impact of the different types of exercises and intensities on such biomarkers, as well as the impact of each recovery method can have on them. It also shows the need for more studies with salivary markers as they have only been evaluated in a few studies.

## Figures and Tables

**Table 1:** PEDro scale analysis.

	PEDro Scale	Rates of Meeting Criteria	
		n	%
1.	Eligibility criteria specified	12	85.7
2.	Random allocation to groups	2	14.3
3.	Concealed allocation	2	14.3
4.	Groups similar at baseline	13	92.8
5.	Blinded subjects	0	0
6.	Blinded therapists/training supervisors	0	0
7.	Blinded evaluators	0	0
8.	Adequacy of follow-up	14	100
9.	Intention-to-treat analysis	11	78.6
10.	Comparison between groups	10	71.4
11.	Measures of variability and point measures	14	100

**Table 2:** Analysis of included studies. C: cortisol; T: testosterone; T:C: testosterone/cortisol ratio; AA: alpha-amylase; IgA: immunoglobulin A; IgG: immunoglobulin G; Mel: melatonin; M: male; F: female; CG: control group; MVC: maximum voluntary contraction; WBH: whole body cryotherapy; ECP: external counter pulsation; CWI: cold water immersion; MMA: mixed martial arts; SSG: small-sided game RES: resistance training; and sec: seconds.

STUDY	POPULATION	OBJECTIVE	INTERVENTION	COMPARISON RECOVERY/METHODS	SALIVA SAMPLES AND ANALYSIS	RESULTS
Cernych 2019	16 M healthy and active (24 ± 4 y)	Evaluate the residual effects of hyperthermia after 2 h recovery	120 s ankle plantar flexion (MVC in isokinetic dynamometer)	Compared control (Passive) and whole-body hyperthermia (WBH—80–90 °C; 30% humidity; 15 min + 3 × 10 min with 15 min rest between sets)	Cortisol (C) Before and 2 h post-recovery	Control: Significantly lower post-recovery (*p* < 0.05) WBH: No significant change
Chiodo 2011	Taekwondo black belt athletes 10 M (14 ± 0 y) 6 F (13 ± 1 y)	Investigate possible stress-related	Youth Taekwondo Competition	Between moments and gender. (No recovery methods described)	Cortisol (C) α Amylase (AA) Morning (9 a.m.), before (15 min) and post-match and recovery phase (30 and 90 min)	C: F = 8.74; *p* = 0.01; ES = 0.37 (gender); F = 0.45; *p* < 0.0001 (sampling). Post hoc: female: differences between peak and other values (*p* range 0.005 to 0.0001 and ES range 0.48 to 0.67). Male: differences between peak and before and post-match and 90 min (*p* range 0.01 to <0.0001 and ES range 0.53 to 0.82) and between 90 min and morning (*p* = 0.003; ES = 0.49) and post-match (*p* = 0.01; ES = 0.56) AA: No difference between genders. Main effect F = 7.33; *p* < 0.0001 (peak value 277.8 ± 45.2 U/mL). Post hoc differences ranged from 0.007 to <0.0001.
Collins 2019	21 M club level athletes (rugby, soccer, Gaelic games, basketball, and hockey) (21.6 ± 3.4 y)	Effects of external counter pulsation (ECP) applied as recovery method	High-intensity exercise (HIE): 1 cycle = CMJ + 20-metre go and 20-metre back + CMJ, with 30 s interval between cycles. Maximum possible cycles until exhaustion or interruption criteria	Compared ECP and rest (CG—passive using ECP without cuff inflation)	Cortisol (C) α Amylase (AA) Testosterone (T) Immunoglobulin A (IgA) T:C ratio Pre, post and 24 h	C: Sig interaction effects F = 4.07; *p* < 0.05. CG: greater at post compared to pre and 24 h (both *p* < 0.001, d = 1.85 and 1.71, respectively). ECP: post greater than 24 h (*p* < 0.0001; d = 1.19). AA: Main effect for time F = 10.05; *p* < 0.001. Greater at post compared to pre and 24 h (both *p* < 0.01, d = 0.29 and 0.33, respectively). No main effect for time when adjusted for secretion rate. Main effects groups (raw and secretion rate) F = 6.52; *p* < 0.05 and F = 5.68; *p* < 0.05, respectively. T: Main effect for time F = 15.02; *p* < 0.001. Greater at post compared to pre and 24 h (both *p* < 0.001, d = 1.44 and 0.62, respectively). IgA: Sig interaction effects F = 4.85; *p* < 0.05. CG: no changes. ECP: greater at post compared to pre and 24 h (both *p* < 0.05, d = 1.26 and 0.86, respectively). T:C ratio: Sig interaction effects F = 4.54; *p* < 0.05. CG: lower at post compared to pre and 24 h (both *p* < 0.05, d = 0.79 and 0.31, respectively). ECP: post lower than 24 h (*p* < 0.05; d = 3.09).
Dimitriou 2015	20 Marathon runners CJ: 7 M 3 F (37 ± 13 y) PL: 6M 4F (38 ± 5 y)	Recovery effects of cherry juice blend intake and placebo	18 athletes in 2008 London Marathon and 2 athletes in West London 2 weeks later with same conditions	Compared cherry juice blend (CJ) and placebo (PL)	Cortisol (C) Immunoglobulin A (IgA) Immunoglobulin G (IgG) Day before, post, 24 and 48 h	C: Sig time effect post compared to the day before (F = 26.291, *p* < 0.001, η_p_^2^ = 0.594) and returned. to baseline at 24 h. No treatment or interaction effects. IgA: No time or interactions effects to IgA concentration. Time effect to output (F = 7.560; *p* < 0.001; η_p_^2^ = 0.296) and decrease post-race in both groups. No treatment or interaction effects (*p* > 0.05) IgG: no time, treatment, or interaction effects.
Hough 2021	23 M healthy and active (21 ± 3 y)	Reliability of the responses of salivary cortisol and testosterone to repeated bouts across several days	3 trials (separated by 7 d). Each one with 30 min in cycle ergometer alternating 1 min at 55% Wmax (maximum work rate) and 4 min at 70% Wmax	Between moments (no recovery methods described)	Cortisol (C) Testosterone (T)	C: Sig time effect (F = 13.949; *p* < 0.001). Sig increases pre to post (*p* = 0.01) and returning to baseline post 30 min (*p* = 0.79). Delta cortisol were similar over the 3 trials (F = 0.680; *p* = 0.518) and found a reliability in the responses to the exercise (ICC = 0.89). T: Sig time effect (F = 70.914; *p* < 0.001). Sig increases pre to post (*p* = 0.001) and post 30 min (*p* < 0.001). Delta testosterones were similar over the 3 trials (F = 2.123; *p* = 0.144).
Lindsay 2015	11 M senior division rugby players	Identify the changes in inflammation following a rugby game and to be used to manage player recovery	Elite amateur rugby game	Between moments. (No recovery methods described)	Cortisol (C) Immunoglobulin A (IgA) Pre-game (24 h), immediately post and 17, 25, 38, 62 and 86 h post-game	C: Sig increase at post-game (*p* = 0.002; η^2^ = 0.583) from pre (15.2 ± 7.2 µmol·L^−1^) to post (60.5 ± 24.6 µmol·L^−1^). Returned to baseline within 17 h. IgA: No change (409 ± 223 to 414 ± 255 µg·mL^−1^) Secretion rate: No change (419 ± 383 to 394 ± 330 µg·min^−1^).
Lindsay 2017	15 semi-professional MMA athletes (28.3 ± 5.7 y)	Effect of cold-water immersion (CWI) in physiological stress parameters	MMA contest-preparation training session	Compared CWI (10 °C whole body) and passive recovery-CG (seated)	Cortisol (C) 7 days prior, pre, immediately post and 1, 2 and 24 h post	General: Sig increase 7 days prior to pre (*p* < 0.05; *d* = 1.18) and pre to post (*p* < 0.05; *d* = 1.23) and to 1 h (*p* < 0.01; *d* = 1.44) (Peak). Maintaining elevated at 2 h (*p* = 0.06; *d* = 0.76) and returned to normality at 24 h. Recovery comparison: CG greater at 2 h (*p* < 0.05; *d* = 0.68); similar in the rest of moments.
McLellan 2011	17 M elite rugby players (19 ± 1.3 y)	Examine responses of rugby league match play	Rugby League match play (and training week continuity) PS: maintenance of daily activities and recovery practice	Between moments. (No recovery methods described)	Cortisol (C) 24 h pre, 30 min pre, 30 min post and 24, 48, 72, 96 and 120 h post	C: Sig higher 30 min pre compared to 24 h pre (*p* = 0.043). Sig increases 30 min post (*p* < 0.001) and 24 h post (*p* < 0.000) to 24 h pre. Sig lower 96 h post to 24 h pre (*p* = 0.042).
Robey 2013	11 M cyclists and triathletes (26 ± 4.4 y)	Effects of high-intensity evening exercise, followed by CWI or not, influenced subsequent sleep	Intensive cycling: 10 min warm up + 15 min at 75% peak power + 5 min break + 15 min maximal time trial	Compared (1) just exercise, (2) control—no exercise or CWI (1 and 2–15 min seated) and (3) exercise + CWI recovery (seated 15 min with 14 °C immersed to the midsternal level	Melatonin (Mel) Pre, 1 h, 2.5 and 10 h post	Main effect for time: *p* < 0.001; main effect for condition: *p* = 0.96; main effect for time X condition: *p* = 0.84. All conditions increased from baseline (~4.6 pM) to 1 h (~23.5 pM) and 2.5 h post (~40 pM), and still elevated 10 h post (~23 pM).
Russell 2017	14 M professional academy soccer players from an English Premier League club (18 ± 2 y)	Effects of single whole-body cryotherapy (WBC)	Repeated sprints: 15 × 30 m timed (60 s interval)	Compared WBC (whole body cryotherapy) and passive recovery—PR (seated)	Cortisol (C) Testosterone (T) Testosterone/cortisol ratio (T:C) Pre, post and 2 h and 24 h post	C: Not differ in trial (Trial × Treatment—F = 0.253; *p* = 0.859; η^2^ = 0.019) and differ in time of sample (F = 13.998; *p* < 0.001; η^2^ = 0.518). Post was similar to pre (*p* = 0.052) and sig decrease at 2 h (*p* = 0.003) and disappear at 24 h. T: Influenced by trial (Trial × Treatment—F = 6.231; *p* = 0.001; η^2^ = 0.326) and time of sample (F = 6.275; *p* = 0.001; η^2^ = 0.326). Similar between trial at pre and post. WBC greater at 2 h (32.5 ± 32.3 pg·mL^−1^) and 24 h (50.4 ± 48.9 pg·mL^−1^) (both *p* = 0.002) T:C: Not differ by trial (Trial × Treatment—F = 0.696; *p* = 0.560; η^2^ = 0.051) but differ in time of sample (F = 8.66; *p* = 0.001; η^2^ = 0.518). Post hoc unable to find differences to pre values.
Shearer 2015	12 M elite rugby players (24.91 ± 4.35 y)	BAM as quick measure of assessing recovery status compared with physiological recovery markers	Rugby domestic league match	Between moments. (No recovery methods described)	Cortisol (C) Testosterone (T) Pre (36 h before), 12, 36 and 60 h post	Sig time effect C:F = 4.9; *p* = 0.01; η^2^ = 0.31 T: F = 3.34; *p* = 0.03; η^2^ = 0.23 Compared from baseline (mean diff; 95% CI) C: 12 h (−0.15; −0.221/−0.079); 36 h (−0.21; −0.364/−0.069) and 60 h (−0.11; −0.254/0.018). T: 12 h (57.34; 16.803/97.884), 36 h (41.31; 3.864/78.763) and 60 h (18.86; −26.268/64.003).
Sparkes 2020	14 M semi-professional soccer players (22.1 ± 3.1 y)	Effects of training order responses a double training day	2 orders training: (1) SSG + RES and (2) RES + SSG	Compared the responses of two orders of training in “recovery” and over 24 h	Cortisol (C) Testosterone (T) Testosterone/Cortisol ratio (T:C) Pre, post and 2 and 24 h post	C: Sig time effect (F = 11.665; *p* = 0.000); no sig interaction (F = 0.814; *p* = 0.494). RES + SSG concentration was greater at 2 h to Pre (*p* = 0.001). T: Sig time effect (F = 5.471; *p* = 0.003); sig interaction (F = 5.196; *p* = 0.004). Concentration differs between protocols at pre to post (*p* = 0.010) and SSG + RES was greater at 2 h to Pre (*p* = 0.001). T:C: Sig time effect (F = 15.333; *p* = 0.000); no sig interaction (F = 0.877; *p* = 0.462). SSG + RES was greater at 2 h (*p* = 0.001).
VanBruggen 2011	12 M trained (22 ± 5 y)	Salivary cortisol responses as effect of different exercise intensity	30 min exercise bouts at 40% (low), 60% (moderate) and 80% (high) of VO2_max_	Compared the responses to different exercise intensities and compared serum and salivary	Cortisol (C) Pre, post and 30 min post	No sig differences in low and mod intensities. In high, sig increase from pre to post (*p* = 0.005) and 30 min (*p* = 0.007). Serum vs. Salivary: significative correlation (Rc = 0.728; *p* = 0.001).
Williams 2018	24 M academy rugby players (21.8 ± 3 y)	Effects of vascular occlusion on recovery	6 × 50 m sprints	Compared vascular occlusion (2 × 3 min with 3 min interval) and shame (cuff at 15 mmHg) recovery	Cortisol (C) Testosterone (T) Testosterone/cortisol ratio (T:C) Pre, post, 2 h and 24 h post	C: Decrease (F = 32.651; *p* < 0.001) and declined over time (F = 7.806; *p* < 0.001) but did not affect recovery (F = 0.640; *p* = 0.531). No diff between conditions (*p* = 0.679). T: Increase (F = 20.127; *p* < 0.001) but did not affect recovery (F = 2.159; *p* = 0.114). No diff between conditions (*p* = 0.226). T:C: Increase (F = 19.200; *p* < 0.001) but did not affect recovery (F = 0.299; *p* = 0.759). No diff between conditions (*p* = 0.421).
